# Evaluation of the Toxicity of *Satureja intermedia* C. A. Mey Essential Oil to Storage and Greenhouse Insect Pests and a Predator Ladybird

**DOI:** 10.3390/foods9060712

**Published:** 2020-06-02

**Authors:** Asgar Ebadollahi, William N. Setzer

**Affiliations:** 1 Moghan College of Agriculture and Natural Resources, University of Mohaghegh Ardabili, Ardabil 56199-36514, Iran; 2 Department of Chemistry, University of Alabama in Huntsville, Huntsville, AL 35899, USA; 3 Aromatic Plant Research Center, 230 N 1200 E, Suite 100, Lehi, UT 84043, USA

**Keywords:** *Aphis nerii*, *Coccinella septempunctata*, plant-based insecticide, *Oryzaephius surinamensis*, *Rhyzopertha dominica*, *Tribolium castaneum*, *Trogoderma granarium*

## Abstract

The use of chemical insecticides has had several side-effects, such as environmental contamination, foodborne residues, and human health threats. The utilization of plant-derived essential oils as efficient bio-rational agents has been acknowledged in pest management strategies. In the present study, the fumigant toxicity of essential oil isolated from *Satureja intermedia* was assessed against cosmopolitan stored-product insect pests: *Trogoderma granarium* Everts (khapra beetle), *Rhyzopertha dominica* (Fabricius) (lesser grain borer), *Tribolium castaneum* (Herbst) (red flour beetle), and *Oryzaephilus surinamensis* (L.) (saw-toothed grain beetle). The essential oil had significant fumigant toxicity against tested insects, which positively depended on essential oil concentrations and the exposure times. Comparative contact toxicity of *S. intermedia* essential oil was measured against *Aphis nerii* Boyer de Fonscolombe (oleander aphid) and its predator *Coccinella septempunctata* L. (seven-spot ladybird). Adult females of *A. nerii* were more susceptible to the contact toxicity than the *C. septempunctata* adults. The dominant compounds in the essential oil of *S. intermedia* were thymol (48.1%), carvacrol (11.8%), *p*-cymene (8.1%), and γ-terpinene (8.1%). The high fumigant toxicity against four major stored-product insect pests, the significant aphidicidal effect on *A. nerii*, and relative safety to the general predator *C. septempunctata* make terpene-rich *S. intermedia* essential oil a potential candidate for use as a plant-based alternative to the detrimental synthetic insecticides.

## 1. Introduction

The Khapra Beetle {*Trogoderma granarium* Everts (Coleoptera: Dermestidae)}, lesser grain borer {*Rhyzopertha dominica* (Fabricius) (Coleoptera: Bostrichidae)}, red flour beetle {*Tribolium castaneum* (Herbst) (Coleoptera: Tenebrionidae)}, and saw-toothed grain beetle {*Oryzaephilus surinamensis* (L.) (Coleoptera: Silvanidae)} are among the most well-known and economically-important stored-product pests with world-wide distribution. Along with direct damage due to feeding on various stored products, the quality of products is strictly diminished because of their residues and mechanically associated microbes [1,2,3,4,5].

Oleander aphid {*Aphis nerii* Boyer de Fonscolombe (Hemiptera: Aphididae)}, as a cosmopolitan obligate parthenogenetic aphid, is a common insect pest of many ornamental plants comprising several species of Asclepiadaceae, Apocynaceae, Asteraceae, Convolvulaceae, and Euphorbiaceae, especially in greenhouse conditions. Along with direct damage, *A. nerii* is able to transmit pathogenic viruses to many plants [6,7,8]. The seven-spot ladybird beetle {*Coccinella septempunctata* L. (Coleoptera: Coccinellidae)} is a natural enemy of various soft-bodied pests like aphids, thrips, and spider mites, and is considered an important biocontrol agent for greenhouse crops [9,10,11].

The utilization of chemical insecticides is the main strategy in the management of insect pests. However, there is a global concern about their numerous side effects including environmental pollution, insecticide resistance, resurgence of secondary pests, and toxicity to non-target organisms ranging from soil microorganisms to pollinator, predator and parasitoid insects, fish, and even humans [12,13,14]. Therefore, the search for eco-friendly and efficient alternative agents for insect pest management is urgent.

Based on the low toxicity to mammals, rapid biodegradation in the environment, and very low chance of insect pest resistance, the use of essential oils extracted from different aromatic plants has been the motivating subject of many researchers in pest management strategies over the past decade [15,16,17,18].

Sixteen species of the *Satureja* genus from the Lamiaceae have been reported in the Iranian flora, of which *S. atropatana* Bunge, *S. bachtiarica* Bunge, *S. edmondi* Briquet, *S. intermedia* C. A. Mey, *S. isophylla* Rech., *S. kallarica* Jamzad, *S. khuzistanica* Jamzad, *S. macrosiphonia* Bornm., *S. sahendica* Bornm., and *S. rechingeri* Jamzad are endemic to Iran [19]. *S. intermedia*, as a small delicate perennial plant growing on rock outcrops, is among aromatic plants with considerable amount (1.45% (*w/w*)) of essential oil [20]. The essential oil of *S. intermedia* is rich in terpenes such as 1,8-cineole, *p*-cymene, limonene, γ-terpinene, α-terpinene, thymol, and β-caryophyllene, which are classified in four main groups; monoterpene hydrocarbons, oxygenated monoterpenoids, sesquiterpene hydrocarbons, and oxygenated sesquiterpenoids [20,21,22]. Some important biological effects of *S. intermedia* essential oil include antifungal, antibacterial, and antioxidant effects, and cytotoxic effects have been reported in previous studies [21,22,23]. Although the susceptibility of insect pests to the essential oils isolated from some *Satureja* species such as *S. hortensis*, *S. montana* L., *S. parnassica* Heldr. & Sart ex Boiss., *S. spinosa* L., and *S. thymbra* L. was documented in recent years [24,25,26], the insecticidal effects of *S. intermedia* essential oil have not reported yet.

As part of a screening program for eco-friendly and efficient plant-derived insecticides, the evaluation of the fumigant toxicity against four major Coleopteran stored-product insect pests *O. surinamensis*, *R. dominica*, *T. castaneum* and *T. granarium* and the contact toxicity against a greenhouse insect pest *Aphis nerii* of the essential oil of *S. intermedia* was the main objective of the present study. Because of the importance of studying the effects of insecticides on the natural enemies of insect pests, the toxicity of *S. intermedia* essential oil against *C. septempunctata* was also investigated.

## 2. Materials and Methods

### 2.1. Plant Materials and Essential Oil Extraction

Aerial parts (3.0 kg) of *S. intermedia* were gathered from the Heiran regions, Ardebil province, Iran (38°23′ N, 48°35′ E, elevation 907 m). It was identified according to the keys provided by Jamzad [27]. The voucher specimen was deposited in the Department of Plant Production, Moghan College of Agriculture and Natural Resources, Ardabil, Iran. The fresh leaves and flowers were separated and dried under shade within a week. One hundred grams of the specimen were poured into a 2-L round-bottom flask and subjected to hydrodistillation using a Clevenger apparatus for 3 h. The extraction was repeated in triplicate and the obtained essential oil was dried over anhydrous Na_2_SO_4_ and stored in a refrigerator at 4 °C.

### 2.2. Essential Oil Characterization

The chemical profile of the *S. intermedia* essential oil was evaluated using gas chromatography (Agilent 7890B) coupled with mass-spectrometer (Agilent 5977A). The analysis was carried out by a HP-5 ms capillary column (30 m × 0.25 mm × 0.25 µm). The temperature of the injector was 280 °C and the column temperature adjusted from 50 to 280 °C using the temperature program: 50 °C (hold for 1 min), increase to 100 °C at 8°/min, increase to 185 °C at 5°/min, increase to 280 °C at 15°/min, and hold at 280 °C for 2 min. The carrier gas was helium (99.999%) with flow rate of 1 mL/min. Essential oil was diluted in methanol, and 1 µL solution was injected (split 1:10 at 0.75 min). The identification of components was performed by comparing mass spectral fragmentation patterns and retention indices with those reported in the databases [28,29,30].

### 2.3. Insects

The required colonies of *Oryzaephilus surinamensis* and *Rhyzopertha dominica* were reared on wheat grains for several generations at the Department of Plant Production, Moghan College of Agriculture and Natural Resources, University of Mohaghegh Ardabili (Ardabil province, Iran). *Tribolium castaneum* and *Trogoderma granarium* adults were collected from infested stored wheat grains in Moghan region (Ardabil province, Iran). Insects were identified by Asgar Ebadollahi. Fifty unsexed pairs of adult insects were separately released onto wheat grains and removed from breeding container after 48 h. Wheat grains contaminated with insect eggs were separately kept in an incubator at 25 ± 2 °C, 65 ± 5% relative humidity and a photoperiod of 14:10 (L:D) h. Finally, one to fourteen-day-old adults of *O. surinamensis*, *R. dominica*, *T. castaneum* and *T. granarium* were designated for fumigant bio-assays.

*Aphis nerii* and its natural predator *Coccinella septempunctata* were used to evaluate the contact toxicity of the *S. intermedia* essential oil. Cohorts of apterous adult females of *A. nerii* and unsexed adults of *C. septempunctata* were taken directly from homegrown oleander (*Nerium oleander* L.) and a chemically untreated alfalfa (*Medicago sativa* L.) field (Moghan region, Ardabil province, Iran), respectively.

### 2.4. Fumigant Toxicity

The fumigant toxicity of *S. intermedia* essential oil was tested on adults of *O. surinamensis*, *R. dominica*, *T. castaneum*, and *T. granarium*. To determine the fumigant toxicity of the essential oil, filter papers (Whatman No. 1, 2 × 2 cm) were impregnated with essential oil concentrations and were attached to the under surface of the screw cap of glass containers (340-mL) as fumigant chambers. A series of concentrations (4.71–14.71, 7.06–20.88, 20.59–58.82, and 8.82–35.29 µL/L for *O. surinamensis*, *R. dominica*, *T. castaneum*, and *T. granarium*, respectively) was organized to assess the toxicity of *S. intermedia* essential oil after an initial concentration setting experiment for each insect species. Twenty unsexed adults (1–14 days old) of each insect species were separately put into glass containers and their caps were tightly affixed. The same conditions without any essential oil concentration were used for control groups and each treatment was replicated five times. Insects mortality was documented 24, 48 and 72 h after initial exposure to the essential oil. Insects were considered dead when no leg or antennal movements were observed [31].

### 2.5. Contact Toxicity

The contact toxicity of *S. intermedia* essential oil against the apterous adult females of *A. nerii* and unsexed adults of *C. septempunctata* was tested through filter paper discs (Whatman No. 1), 9 cm diameter, positioned in glass petri dishes (90 × 10 mm). Range-finding experiments were established to find the proper concentrations for each insect. Concentrations ranging from 200 to 750 µg/mL for *A. nerii* and from 500 to 1400 µg/mL for *C. septempunctata* were prepared via 1.00% aqueous Tween-80 as an emulsifying agent. Each solution (200 µL) was applied to the surface of the filter paper. Ten insects were separately released onto each treated disc, the dishes sealed with Parafilm^®^ and kept at 25 ± 2 °C, 65 ± 5% relative humidity and a photoperiod of 16:8 h (light:dark). Except for the addition of essential oil concentrations, all other procedures were unchanged for the control groups. Four replications were made for each treatment and mortality was documented after 24 h. Aphids and ladybirds were considered dead if no leg or antennal movements were detected when softly prodded [32,33].

### 2.6. Data Analysis

The mortality percentage was corrected using Abbott’s formula: *P*t = [(*P*o − *P*c)*/*(100 − *P*c)] × 100, in which *P*t is the corrected mortality percentage, *P*o is the mortality (%) caused by essential oil concentrations and *P*c is the mortality (%) in the control groups [34].

Analysis of variance (ANOVA) and Tukey’s test at *p* = 0.05 were used to statistically identify the effects of independent factors (essential oil concentration and exposure time) on insect mortality and the differences among mean mortality percentage of insects, respectively. Probit analysis was used to estimate LC_50_ and LC_95_ values with 95% fiducial limits, the data heterogeneity and linear regression information using SPSS 24.0 software package (Chicago, IL, USA).

## 3. Results

### 3.1. Chemical Composition of Essential Oil

The chemical composition of *S. intermedia* essential oil is presented in Table 1. A total of 47 compounds were identified in the essential oil, in which the phenolic monoterpenoids thymol (48.1%) and carvacrol (11.8%), along with *p*-cymene (8.1%), γ-terpinene (8.1%), carvacryl methyl ether (4.0%), α-pinene (2.7%), and β-caryophyllene (2.4%) were dominants. Terpenoids were the most abundant components (98.6%), especially monoterpene hydrocarbons (20.5%) and oxygenated monoterpenoids (68.4%) with only minor amounts of phenylpropanoids or fatty acid-derived compounds.

### 3.2. Fumigant Toxicity

**Figure 1:**
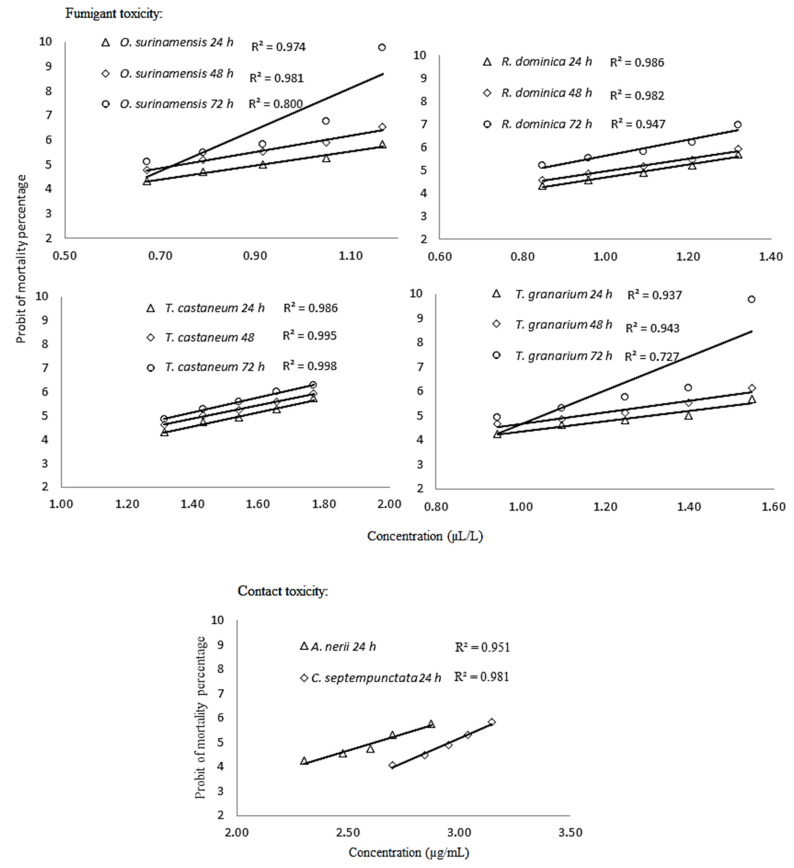
Concentration–response lines of contact and fumigant toxicity of Satureja intermedia essential oil against Aphis nerii and Coccinella septempunctata, and Oryzaephilus surinamensis, Rhyzopertha dominica, Tribolium castaneum, and Trogoderma granarium, respectively.

Analysis of variance (ANOVA) revealed that the tested concentrations of *S. intermedia* essential oil (*F* = 239.462 and *p* < 0.0001 for *O. surinamensis*, *F* = 223.629 and *p* < 0.0001 for *R. dominica*, *F* = 169.615 and *p* < 0.0001 for *T. castaneum*, and *F* = 89.032 and *p* < 0.0001 for *T. granarium* with df = 4, 45) and the considered exposure times (*F* = 212.855 and *p* < 0.0001 for *O. surinamensis*, *F* = 281.180 and *p* < 0.0001 for *R. dominica*, *F* = 84.705 and *p* < 0.0001 for *T. castaneum*, and *F* = 84.501 and *p* < 0.0001 for *T. granarium* with df = 2, 45) had significant effects on the mortality of all insect pests. According to Figure 1 and relatively high *R*^2^ values, there is a positive correlation between the fumigation of essential oil concentrations and the mortality of four storage insect pests at all exposure times. Furthermore, the steep slopes indicate a homogenous toxic response among beetles to the essential oil.

According to Table 2, an obvious difference in the mean mortality percentage of all tested storage insect pests was detected, as essential oil concentration and exposure time were increased. For example, 25.00% mortality of *O. surinamensis* adults was observed at 4.71 µL/L and 24-h exposure time, which had increased to 80.00% and 100% at 14.71 µL/L after 24 and 72 h, respectively. It is apparent that the essential oil of *S. intermedia* gave at least 90% mortality against all tested stored-product insect pests at 58.82 µL/L after 72 h (Table 2).

Based on lower LC_50_ values of those stored-product insect pests tested, *O*. *surinamensis* was significantly the most susceptible insect to the essential oil of *S. intermedia* at all time intervals. In contrast, the adults of *T. castaneum* with highest LC_50_ and LC_95_ values were the most tolerant to fumigation with *S. intermedia* essential oil. Furthermore, the susceptibility of insect pests to the fumigation of *S. intermedia* essential oil followed in the order: *O. surinamensis* > *R. dominica* > *T. granarium* > *T. castaneum* (Table 3).

### 3.3. Contact Toxicity

The tested concentrations of *S. intermedia* essential oil demonstrated significant contact toxicity on both *A. nerii* (*F* = 27.682, df = 4, 15; *p* < 0.0001) and *C. septempunctata* (*F* = 35.607, df = 4, 15; *p* < 0.0001). A positive correlation between essential oil concentrations and the mortality of *A. nerii* and *C. septempunctata* in the contact assay is also apparent, based on the high *R*^2^ values (Figure 1). Comparisons of the mean mortality percentage of *A. nerii* and its predator *C. septempunctata* caused by *S. intermedia* essential oil are shown in Table 4. The mortality percentages of both insects increased with increasing essential oil concentrations, but their susceptibility to the essential oil was noticeably different. For example, 62.50% mortality was documented for *A. nerii* at 500 µg/mL essential oil concentration while its predator *C. septempunctata* was more tolerant and exhibited only 17.50% mortality at this concentration (Table 4).

The results of the probit analysis for the contact toxicity of *S. intermedia* essential oil against *A. nerii* and *C. septempunctata* adults are shown in Table 5. According to low LC_50_ and LC_95_ values, the adult females of *A. nerii* were more susceptible to contact toxicity of *S. intermedia* essential oil than the adults of *C. septempunctata*.

## 4. Discussion

The susceptibility of *O. surinamensis*, *R. dominica*, *T. castaneum* and *T. granarium* adults to the essential oil of *S. intermedia* with 24-h LC_50_ values of 8.151, 12.825, 20.489, and 35.612 µL/L, respectively, was distinguished in the present study. The fumigant toxicity of some plant-derived essential oils against *O. surinamensis*, *R. dominica*, *T. castaneum* and *T. granarium* has been documented in previous studies; it was found that the essential oils of *Agastache foeniculum* (Pursh) Kuntze, *Achillea filipendulina* Lam., and *Achillea millefolium* L. with respective 24-h LC_50_ values of 18.781, 12.121, and 17.977 µL/L, had high toxicity on the adults of *O. surinamensis* [31,34,35,36]. The adults of *R. dominica* were also susceptible to the fumigation of essential oils extracted from *Eucalyptus globulus* Labill (24-h LC_50_ = 3.529 μL/L), *Lavandula stoechas* L. (24-h LC_50_ = 5.660 μL/L), and *Apium graveolens* L. (24-h LC_50_ = 53.506 μL/L) [37,38]. The fumigation of the essential oils of *Lippia citriodora* Kunth (24-h LC_50_ = 37.349 μL/L), *Melissa officinalis* L. (24-h LC_50_ = 19.418 μL/L), and *Teucrium polium* L. (24-h LC_50_ = 20.749 μL/L) resulted in significant mortality in *T. castaneum* [39,40,41]. The essential oils of *Schinus molle* L. (48-h LC_50_ = 806.50 μL/L) and *Artemisia sieberi* Besser (24-h LC_50_ = 33.80 μL/L) also had notable fumigant toxicity against the adults of *T. granarium* [42,43]. The toxicity of all the above-mentioned essential oils was augmented when the exposure time was prolonged. These findings support the results regarding the time-dependent susceptibility of *O. surinamensis*, *R. dominica*, *T. castaneum* and *T. granarium* to plant essential oils. The differences in observed LC_50_ values are likely due to the differences in the essential oil compositions from the different plant species and possibly to differences in the experimental conditions. Furthermore, the *S. intermedia* essential oil with low 24-h LC_50_ value was more toxic on *O. surinamensis* than *A. foeniculum*, *A. filipendulina*, and *A. millefolium* essential oils, on *R. dominica* than *A. graveolens* essential oil, on *T. castaneum* than *Lippia citriodora* essential oil, and on *T. granarium* than *S. molle* essential oil.

The terpenes, especially thymol, carvacrol, *p*-cymene and γ-terpinene, were recognized as the main components of *S. intermedia* essential oil in the present study. In the study of Sefidkon and Jamzad, thymol (32.3%), γ-terpinene (29.3%), *p*-cymene (14.7%), elemicin (4.8%), limonene (3.3%), and α-terpinene (3.3%) were the main components of *S. intermedia* essential oil [20]. In another study, thymol (34.5%), γ-terpinene (18.2%), *p*-cymene (10.5%), limonene (7.3%), α-terpinene (7.1%), carvacrol (6.9%), and elemicin (5.3%) were found to be major components in the essential oil of *S. intermedia* [23]. In the present study, however, limonene was a minor component (0.5%), and neither elemicin nor α-terpinene were detected. Ghorbanpour et al. reported the terpenes thymol (32.3%), *p*-cymene (14.7%), γ-terpinene (3.3%), and carvacrol (1.0%), and the phenylpropanoid elemicin (4.8%) as the main components in the essential oil of *S. intermedia* [22], while the concentrations of γ-terpinene and carvacrol were much lower compared to the present findings. The differences in the chemical profile of the plant essential oils are likely due to the internal and external factors such as seasonal variation, geographical features, plant growth stage, and different extraction conditions [19,44,45]. The insecticidal properties of several terpenes, especially monoterpene hydrocarbons and monoterpenoids, which accounted for 88.9% of the *S. intermedia* essential oil in the present study, have been documented in recent investigations. For example, insecticidal activities of *p*-cymene, α-pinene, γ-terpinene, 1,8-cineole, and limonene have been demonstrated against several detrimental insect pests [46,47,48,49,50]. Previous studies have also indicated that the monoterpenoids thymol and carvacrol had significant toxicity against insect pests [46,51,52]. Accordingly, the insecticidal efficiency of *S. intermedia* essential oil can be attributed to such components.

The contact toxicity of the essential oil of *Eucalyptus globulus* Labill. against *A. nerii* has been reported by Russo et al. [53]. Although this is the only previous study to investigate the susceptibility of *A. nerii* to a plant essential oil, its findings confirm the results of the present study about the possibility of *A. nerii* management through plant essential oils. Indeed, the toxicity of *S. intermedia* essential oil was evaluated for the first time in the present study against *A. nerii* and its natural enemy *C. septempunctata*. The essential oil of *S. intermedia* was more toxic on *A. nerii* (LC_50_: 418 µg/mL) than the predator ladybird *C. septempunctata* (LC_50_: 914 µg/mL), suggesting that the predator was more tolerant than the aphid to *S. intermedia* essential oil, which is very valuable in terms of predator protection. Similar results were obtained for controlling aphids [54,55] and some other insect pests [56,57,58] using plant-derived essential oils along with protecting their predators. However, the destructive side-effects of some essential oils on parasitoids have been reported [59,60,61]. Therefore, it is important to select efficient pesticides with lower side effects on natural enemies at operative concentrations to the pests, which has been achieved in the current study.

## 5. Conclusions

In conclusion, the terpene-rich essential oil of *S. intermedia* has significant fumigant toxicity against the adults of *O. surinamensis*, *R. dominica*, *T. castaneum*, and *T. granarium*, and may be considered as a natural effective fumigant on stored products. This bio-rational agent also has significant contact toxicity on the adult females of *A. nerii*, one of the cosmopolitan insect pests of ornamental plants. Furthermore, the predator ladybird *C. septempunctata* was more tolerant to the essential oil than the aphid. Accordingly, *S. intermedia* essential oil can be nominated as an eco-friendly efficient insecticide by decreasing the risks associated with the application of synthetic chemicals. However, the exploration of any side-effects of the essential oil on other useful insects such as parasitoids and pollinators, its phytotoxicity on the treated plants and crops, any adverse tastes or odors on stored products, and the preparation of novel formulations to increase its stability in the environment for practical utilization are needed.

## Figures and Tables

**Table 1:** Chemical composition of the essential oil isolated from aerial parts of *Satureja intermedia*.

RI_calc_	RI_db_	Compound	%	RI_calc_	RI_db_	Compound	%
929	932	α-Pinene	2.7	1384	1387	β-Bourbonene	0.1
984	974	1-Octen-3-ol	0.3	1389	1379	Geranyl acetate	tr
990	988	Myrcene	0.4	1423	1417	β-Caryophyllene	2.4
1016	1020	*p*-Cymene	8.1	1428	1431	β-Gurjunene	0.1
1034	1024	Limonene	0.5	1432	1442	α-Maaliene	0.1
1037	1026	1,8-Cineole	1.7	1438	1439	Aromadendrene	0.7
1060	1054	γ-Terpinene	8.1	1454	1452	α-Humulene	0.3
1066	1065	*cis*-Sabinene hydrate	0.4	1476	1478	γ-Muurolene	0.5
1083	1086	Terpinolene	0.2	1487	1489	β-Selinene	0.2
1083	1089	*p*-Cymenene	0.2	1496	1496	Viridiflorene	0.7
1092	1095	Linalool	0.2	1500	1500	α-Muurolene	0.2
1094	1098	*trans*-Sabinene hydrate	0.1	1510	1505	β-Bisabolene	1.3
1121	1128	*allo*-Ocimene	0.2	1515	1513	γ-Cadinene	0.3
1164	1165	Borneol	0.4	1523	1522	δ-Cadinene	0.7
1176	1174	Terpinen-4-ol	0.8	1530	1533	*trans*-Cadina-1,4-diene	0.1
1187	1191	Hexyl butyrate	0.1	1535	1537	α-Cadinene	tr
1239	1241	Carvacryl methyl ether	4.0	1540	1544	α-Calacorene	0.3
1284	1282	(*E*)-Anethole	0.7	1557	1553	Thymohydroquinone	0.5
1290	1289	Thymol	48.1	1578	1577	Spathulenol	0.9
1298	1298	Carvacrol	11.8	1581	1582	Caryophyllene oxide	0.8
1340	1340	Piperitenone	tr			Monoterpene hydrocarbons	20.5
1346	1346	α-Terpinyl acetate	0.1			Oxygenated monoterpenoids	68.4
1349	1349	Thymyl acetate	0.2			Sesquiterpene hydrocarbons	8.0
1357	1356	Eugenol	0.1			Oxygenated sesquiterpenoids	1.7
1365	1373	α-Ylangene	0.1			Phenylpropanoids	0.8
1371	1374	α-Copaene	0.2			Others	0.4
1376	1372	Carvacryl acetate	0.1			Total identified	99.8

RI_calc_ = Retention index determined with respect to a homologous series of *n*-alkanes on a HP-5 ms column; RI_db_ = Retention index from the databases [28,29,30]; tr = trace (<0.05%).

**Table 2:** Mean mortality ± SE of the adults of *Oryzaephilus surinamensis*, *Rhyzopertha dominica*, *Tribolium castaneum*, and *Trogoderma granarium* exposed to the fumigation of *Satureja intermedia* essential oil after 24, 48, and 72 h.

Insect	Time (h)	Concentration (µL/L)				
		**4.71**	**6.18**	**8.24**	**11.18**	**14.71**
*O. surinamensis*	24	25.00 ± 0.41 ^j^	38.75 ± 0.63 ^i^	50.00 ± 0.41 ^g^	60.00 ±0.41 ^f^	80.00 ± 0.41 ^d^
	48	41.25 ± 0.48 ^h^	57.50 ± 0.29 ^f,g^	70.00 ± 0.41 ^e^	81.25 ± 0.48 ^d^	93.75 ± 0.48 ^c^
	72	53.75 ± 0.48 ^g^	68.75 ± 0.48 ^e^	80.00 ± 0.58 ^d^	96.25 ± 0.48 ^b^	100.00 ± 0.00 ^a^
		**7.06**	**9.12**	**12.35**	**16.18**	**20.88**
*R. dominica*	24	25.00 ± 0.41 ^l^	33.75 ± 0.48 ^k^	46.25 ± 0.48 ^i^	58.75 ± 0.29 ^h^	75.00 ± 0.58 ^e^
	48	33.75 ± 0.48 ^k^	43.75 ± 0.48 ^j^	56.25 ± 0.48 ^h^	67.50 ± 0.29 ^g^	82.50 ± 0.29 ^c^
	72	57.50 ± 0.29 ^h^	70.00 ± 0.41 ^f^	78.75 ± 0.25 ^d^	88.75 ± 0.48 ^b^	97.50 ± 0.29 ^a^
		**20.59**	**27.06**	**34.71**	**45.29**	**58.82**
*T. castaneum*	24	23.75 ± 0.48 ^k^	38.75 ± 0.48 ^i^	46.25 ± 0.48 ^g^	60.00 ±0.41 ^e^	76.25 ± 0.25 ^c^
	48	35.00 ± 0.58 ^j^	50.00 ± 0.58 ^f^	58.75 ± 0.63 ^e^	71.25 ± 0.48 ^d^	82.50 ± 0.50 ^b^
	72	43.75 ± 0.48 ^h^	60.00 ± 0.41 ^e^	71.25 ± 0.25 ^d^	83.75 ± 0.63 ^b^	90.00 ± 0.50 ^a^
		**8.82**	**12.53**	**17.68**	**25.00**	**35.29**
*T. granarium*	24	22.50 ± 0.48 ^j^	35.00 ± 0.29 ^i^	42.50 ± 0.25 ^h^	50.00 ± 0.41 ^g^	75.00 ± 0.29 ^c^
	48	37.50 ± 0.25 ^i^	45.00 ± 0.29 ^h^	55.00 ± 0.29 ^f^	70.00 ± 0.41 ^d^	87.50 ± 0.48 ^b^
	72	47.50 ± 0.25 ^g^	62.50 ± 0.48 ^e^	77.50 ± 0.48 ^c^	87.50 ± 0.48 ^b^	100.00 ± 0.00 ^a^

Data that do not have the same letters are statistically significant different at *p* = 0.05 based on Tukey’s test. Each datum represents mean ± SE of four replicates with eighty adult insects.

**Table 3:** Probit analysis of the data obtained from fumigation of *Satureja intermedia* essential oil on the adults of *Oryzaephilus surinamensis*, *Rhyzopertha dominica*, *Tribolium castaneum*, and *Trogoderma granarium*.

Insect	Time (h)	LC_50_ with 95% Confidence Limits (µL/L)	LC_90_ with 95% Confidence Limits (µL/L)	χ^2^ (df = 3)	Slope ± SE	Sig. *
*O. surinamensis*	24	8.151 (7.396–8.970)	23.177 (18.675–32.578)	1.99	2.824 ± 0.344	0.574
	48	5.542 (4.853–6.119)	13.710 (11.971–16.756)	1.288	3.258 ± 0.378	0.732
	72	4.716 (4.143–5.174)	9.200 (8.413–10.405)	5.134	4.415 ± 0.504	0.162
*R. dominica*	24	12.825 (11.661–14.189)	36.901 (29.147–54.0970)	0.885	2.792 ± 0.356	0.829
	48	10.398 (9.265–11.454)	30.455 (24.687–42.838)	1.056	2.746 ± 0.358	0.788
	72	6.358 (5.126–7.296)	15.970 (14.160–19.138)	2.488	3.204 ± 0.432	0.477
*T. granarium*	24	20.489 (18.114–23.612)	81.507 (58.604–140.911)	4.233	2.137 ± 0.283	0.237
	48	13.654 (11.811–15.364)	49.192 (38.852–71.499)	3.978	2.302 ± 0.289	0.264
	72	9.785 (6.082–12.258)	24.075 (18.870–42.027)	5.842	3.277 ± 0.360	0.12
*T. castaneum*	24	35.612 (32.538–39.070)	95.948 (77.352–135.744)	0.967	2.977 ± 0.376	0.809
	48	28.048 (24.747–30.916)	80.251 (65.751–111.454)	0.297	2.807 ± 0.378	0.961
	72	22.861 (19.648–25.415)	57.584 (50.068–71.481)	0.139	3.194 ± 0.405	0.987

* Since the significance level is greater than 0.05, no heterogeneity factor is used in the calculation of confidence limits. The number of insects for calculation of LC_50_ values is 200 for *T. granarium* and 400 for other insects in each time.

**Table 4:** Mean mortality ± SE of the adults of *Aphis nerii* and *Coccinella septempunctata* exposed to the different concentration of *Satureja intermedia* essential oil after 24 h.

Insect	Concentration (μg/mL)				
	**200**	**300**	**400**	**500**	**750**
*A. nerii*	22.50 ± 0.25 ^e^	32.50 ± 0.25 ^d^	40.00 ± 0.41 ^c^	62.50 ± 0.25 ^b^	77.50 ± 0.75 ^a^
	**500**	**700**	**900**	**1100**	**1400**
*C. septempunctata*	17.50 ± 0.48 ^e^	30.00 ± 0.41 ^d^	45.00 ± 0.29 ^c^	62.50 ± 0.48 ^b^	80.00 ± 0.41 ^a^

Data that do not have the same letters are statistically significant different at *p* = 0.05 based on Tukey’s test. Each datum represents mean ± SE of four replicates with eighty adult insects.

**Table 5:** Probit analysis of the data obtained from contact toxicity of *Satureja intermedia* essential oil on the adults of *Aphis nerii* and *Coccinella septempunctata*.

Insect	LC_50_ with 95% Confidence Limits (μg/mL)	LC_90_ with 95% Confidence Limits (μg/mL)	χ^2^ (df = 3)	Slope ± SE	Sig. *
*A. nerii*	418.379 (379.586–464.130)	1224.788 (975.704–1738.840)	4.363	2.747 ± 0.318	0.225
*C. septempunctata*	913.722 (853.739–980.799)	1908.099 (1652.748–2352.473)	1.932	4.008 ± 0.413	0.587

* Since the significance level is greater than 0.05, no heterogeneity factor is used in the calculation of confidence limits. The number of insects for calculation of LC_50_ values is 240 for each insect.
